# Evaluation of bile salt hydrolase inhibitor efficacy for modulating host bile profile and physiology using a chicken model system

**DOI:** 10.1038/s41598-020-61723-7

**Published:** 2020-03-18

**Authors:** Wenjing Geng, Sarah L. Long, Yun-Juan Chang, Arnold M. Saxton, Susan A. Joyce, Jun Lin

**Affiliations:** 1 Department of Animal Science, The University of Tennessee, 2506 River Drive, Knoxville, USA; 2 APC Microbiome Ireland, University College Cork, Cork, Ireland; 3 School of Biochemistry and Cell Biology, University College Cork, Cork, Ireland; 4 Department of High Performance Computing and Research, University of Rutgers, Newark, USA

**Keywords:** Bacteria, Metabolic engineering, Gastrointestinal system

## Abstract

Gut microbial enzymes, bile salt hydrolases (BSHs) are the gateway enzymes for bile acid (BA) modification in the gut. This activity is a promising target for developing innovative non-antibiotic growth promoters to enhance animal production and health. Compelling evidence has shown that inhibition of BSH activity should enhance weight gain by altering the BA pool, host signalling and lipid metabolism. We recently identified a panel of promising BSH inhibitors. Here, we address the potential of them as alternative, effective, non-antibiotic feed additives, for commercial application, to promote animal growth using a chicken model. In this study, the *in vivo* efficacy of three BSH inhibitors (caffeic acid phenethylester, riboflavin, carnosic acid) were evaluated. 7-day old chicks (10 birds/group) were either untreated or they received one of the specific BSH inhibitors (25 mg/kg body weight) *via* oral gavage for 17 days. The chicks in treatment groups consistently displayed higher body weight gain than the untreated chicks. Metabolomic analysis demonstrated that BSH inhibitor treatment led to significant changes in both circulating and intestinal BA signatures in support of blunted intestinal BSH activity. Consistent with this finding, liver and intestinal tissue RNA-Seq analysis showed that carnosic acid treatment significantly altered expression of genes involved in lipid and bile acid metabolism. Taken together, this study validates microbial BSH activity inhibition as an alternative target and strategy to antibiotic treatment for animal growth promotion.

## Introduction

Antibiotic growth promoters (AGPs), a panel of different classes of antibiotics delivered in feed at sub-therapeutic doses to target the gut microbiota, have been applied to induce consistent and reproducible weight gain in livestock since the 1940’s^1^. However, evidence of a link between AGP usage and the emergence of antibiotic-resistant microbes, including human pathogens of animal origin, has led to a European ban on AGPs since 2006 so that currently, there is a worldwide trend to limit their applications^2,3^. Thus, developing innovative non-antibiotic technology to replace AGPs is urgently required in order to maintain levels of animal production for food, without impacting public health.

Characterization of the gut microbial community (microbiome) in response to AGP treatment is critical to improve our understanding of the modes of action of AGPs, and for developing non-antibiotic alternatives to AGPs^2,4,5^. One functional aspect that shows enormous potential is bile acid metabolism. Consistently, the growth-promoting effects of AGP has been highly correlated with decreased microbial bile salt hydrolase activity (BSH), a function that can negatively impact host fat digestion and lipid metabolism^4,6^. BSHs catalyze deconjugation of liver released amino conjugated bile acids (BAs) in the intestine and they serve as a class of gateway microbial enzymes controlling downstream microbial and host BA metabolism in the intestine^6,7^. Furthermore, extensive gut microbiome studies have demonstrated that AGP usage significantly reduces microbial populations of powerful BSH-producers in the intestine, including *Lactobacillus* species^4^. Indeed, more recently, *in vivo* evidence demonstrates that manipulation of BSH activity alone could significantly influence lipid metabolism, signaling functions, and weight gain in a murine host^8^.

In light of these findings, we hypothesized that dietary supplementation with BSH inhibitors could alter host lipid metabolism and energy harvest and consequently enhance feed efficiency and body weight gain in animals raised for food supply. We have identified and characterized a unique *Lactobacillus salivarius* BSH enzyme with a broad conjugated BA substrate specificity of chicken gut origin^9^. This BSH was applied for effective high-throughput screening to identify a group of promising BSH inhibitors that could act as an alternative approach to AGPs^10^. Understanding the *in vivo* behavior of these BSH inhibitors is important in order to determine whether their oral administration can facilitate effective transit to the complex gastrointestinal tract and exert inhibitory effects on intestinal BSHs as well as to examine their impact. Examining BA signature changes and their effects on host gene expression in a consumer relevant model, the chicken, will inform the development of BSH inhibitors as effective non-antibiotic feed additives for non-antibiotic growth promotion.

In this proof-of-concept study, the *in vivo* efficacy of three promising BSH inhibitors, riboflavin, caffeic acid phenethylester (CAPE), and carnosic acid^10^, were evaluated using the chicken model system. We performed a cage trial with limited chicken number of 10 birds per group, as opposed to industry-oriented pen trial for comprehensive nutritional measurement, usually >120 birds per treatment group^11^. This study aimed to determine whether feed delivered BSH inhibitors could effectively induce BA changes and alter chicken body weight gain and feed efficiency. It further aimed to determine the influence of one BSH inhibitor, carnosic acid, on local (intestine) and systemic (liver) transcriptome responses in validating inhibitor action.

## Results

### Oral BSH inhibitor delivery revealed responder (RS) and non-responder (NRS) growth promotion when compared to untreated animals

Seven day old chicks were randomly allocated into four groups (n = 10/group). Each group received none (50% propylene glycol control solution) or one of the BSH inhibitors via oral gavage (once a day) for 21 consecutive days. All of the chickens exhibited normal growth behavior and no mortality occurred during the 28 days of the experimental period. No weight loss was evident for any treatment administered relative to control animals (Table 1). In general, oral administration of each BSH inhibitor consistently enhanced overall body weight (BW) and actual BW gain at the different time-points and by 24 days of age (Table 1). However, despite these trends the differences in BW and BW gain were not statistically significant (*P* > 0.05).

**Table 1:** Body weight, feed conversion ratio and body weight gain monitored over time in chickens treated or not with different BSH inhibitors over 21 days.

Chicken Age	Control	CAPE	Riboflavin	Carnosic Acid	*P* value
**Chicken Body Weight (g/bird)**					
Day 7	163.56 ± 3.41	162.57 ± 4.92	164.19 ± 3.27	162.76 ± 3.32	0.99
Day 10	249.42 ± 5.83	244.91 ± 5.12	258.46 ± 4.96	251.58 ± 6.20	0.43
Day 14	421.16 ± 8.33	421.37 ± 9.88	431.98 ± 7.69	436.48 ± 8.87	0.54
Day 17	580.32 ± 10.68	585.68 ± 15.35	611.68 ± 13.50	601.10 ± 14.47	0.40
Day 21	813.11 ± 12.64	829.46 ± 22.95	869.59 ± 22.12	859.72 ± 22.03	0.25
Day 24	967.95 ± 20.89	1022.36 ± 29.85	1053.10 ± 33.74	1053.98 ± 26.77	0.19
Day 28	1234.26 ± 33.55	1294.11 ± 40.51	1304.03 ± 44.99	1293.40 ± 25.76	0.56
**Chicken Body Weight Gain (g/bird/day)**					
Day 7–10	28.62 ± 0.95	27.45 ± 0.49	31.48 ± 0.98	29.58 ± 1.03	0.11
Day 10–14	42.99 ± 0.90	44.15 ± 1.27	43.33 ± 1.59	46.11 ± 1.35	0.48
Day 14–17	53.05 ± 1.97	54.77 ± 2.09	59.90 ± 3.56	54.87 ± 2.74	0.36
Day 17–21	58.20 ± 1.65	60.95 ± 2.55	64.48 ± 2.61	64.66 ± 2.37	0.23
Day 21–24	51.61 ± 3.96	64.30 ± 3.94	61.17 ± 5.43	64.75 ± 3.33	0.18
Day 24–28	66.67 ± 4.25	68.06 ± 4.53	62.78 ± 3.55	59.90 ± 3.28	0.52
**Feed Conversion Ratio (g of feed/ g of weight gain)**					
Day 10–14	1.64 ± 0.03^ab^	1.56 ± 0.04^b^	1.81 ± 0.08 ^a^	1.53 ± 0.05^b^	0.03
Day 14–17	2.03 ± 0.08	1.94 ± 0.09	1.92 ± 0.09	1.91 ± 0.13	0.84
Day 17–21	1.93 ± 0.05	1.82 ± 0.09	1.83 ± 0.08	1.66 ± 0.06	0.21
Day 21–24	2.49 ± 0.24	1.98 ± 0.15	2.15 ± 0.22	1.93 ± 0.11	0.22
Day 24–28	2.21 ± 0.14	2.15 ± 0.21	2.36 ± 0.16	2.31 ± 0.19	0.84

Growth performance data (body weight, body weight gain, and feed conversion ratio) is represented as mean ± standard error. Different letters in the same row represent significant difference between treatments (Tukey, P < 0.05).

Food intake was determined for each group throughout the duration of this study and the feed conversion ratio (FCR), an indicator for feeding efficiency, was recorded (Table 1). Oral administration of each BSH inhibitor consistently led to lower FCR than that in the control group during feeding periods examined: day 14–17, day 17–21, and day 21–24 despite a lack of significant difference (*P* > 0.05) (Table 1). However, FCR was significantly different during the initial period, for BSH inhibitor-treated groups (1.82, 1.85, and 1.75 for riboflavin, CAPE, and carnosic acid, respectively) than that of the control group (2.11).

**Figure 1:**
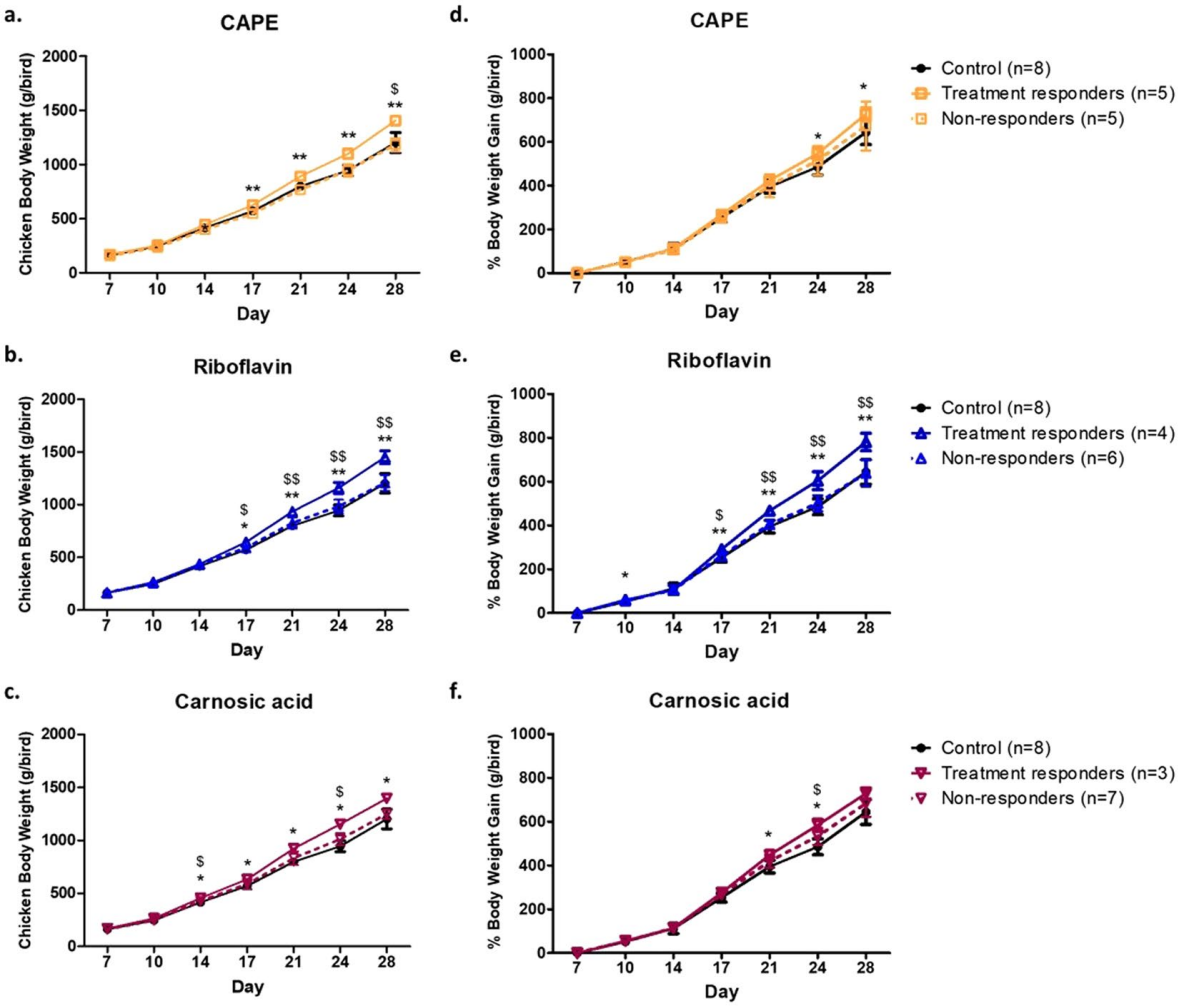
Body weight (BW) gain is influenced by BSH inhibitor treatment. Treatments were sub-divided according to responders (RS) and non-responders (NRS) and graphed relative to untreated control animals. BW gain is shown following treatment with (**a**) CAPE, (**b**) riboflavin and (**c**) carnosic acid via oral gavage for 21 consecutive days. Corresponding values for actual weight gain are shown for (**d**) CAPE, (**e**) riboflavin and (**f**) carnosic acid. Data is represented as mean ± SD.

When individual body weight and weight gain were examined over time following the different treatments, the animals could be stratified into two cohorts relative to the untreated group by principal component analysis (PCA analysis as shown in Fig. 1S). For each treatment non-responders (NRS) clustered with control, untreated animals while responders (RS) diverged and clustered together. On this basis, a threshold value of 1160 g/bird for NRS was calculated and any animal with a final weight above this value were classed as treatment RS. The actual division of animals on this basis for the different treatments are as follow: CAPE: RS n = 5, NRS n = 5; Riboflavin: RS n = 4, NRS n = 6; Carnosic acid: RS n = 4, NRS n = 6. Significant increases in body weight among RS was evident at 14 days for carnosic acid (*P* > 0.05) and at 17 days for both riboflavin and CAPE (*P* > 0.05) Fig. 1a–c. The RS body weight continued to increase significantly for the duration of the trial. The inhibitor potency to improve body mass among RS is as follows: riboflavin > CAPE > carnosic acid. When actual weight gain is measured (Fig. 1d–f), the potency varied in the order of riboflavin > carnosic acid > CAPE among RS. Accurate assessment of feed efficiency by RS and NS could not be performed since food intake was assessed collectively or per cage.

### BSH inhibitor treatment alters BA profiles in the ileum

**Figure 2:**
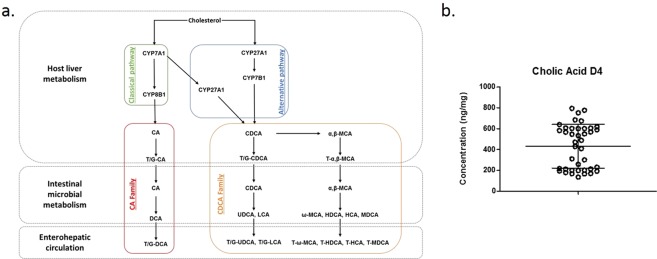
Assessment of Ileum bile acid extraction efficiency (**a**) Schematic of bile acid synthesis and subsequent microbial modifications from liver through the intestine indicating family and other classifications (**b**) Levels of internal standard deuterated cholic acid (D4 CA). Data is expressed as ng/mg of each sample and represented as mean ± SD.

The collective effect of BSH (and its inhibitors) on bile acid signatures can feedback to alter BA synthesis, BA ileal uptake, host signaling locally and systemically, as well as microbial populations. In order to evaluate intestinal BSH activity and its inhibition, we examined both the local ileal and systemic (blood) BA composition in the presence and absence of dietary BSH inhibitors. Both the ileal contents and blood samples were collected from each individual chicken (n = 10) at end point, day 28, whether they were RS or NRS. Extracted samples were subjected to BA analysis following UPLC TMS based targeted analysis of 31 different BA moieties across the BA classes (Fig. 2a). For all samples, quality control spiking of each sample with internal standard deuterated cholic acid (D4 CA) prior to extraction allowed extraction efficiency assessment and BA level normalization. D4 CA recoveries were in an acceptable range indicating efficient and accurate sample extraction for ileal samples (Fig. 2b).

Individual bile acid moieties were quantified against their corresponding standard curve, the data for each moiety is recorded along with any statistical significance relative to the control animals (Tables 2 and 3, respectively). Interestingly, similar to rodents, control chicken ileal bile acid was dominated by tauro conjugated BA representing approximately 30% of total ileal BA while glyco conjugated BA was less well represented at just 0.3%. Primary BA was 100 fold higher than glyco conjugated BA and secondary BA represented 0.1% of the total BAs featured. In collective comparisons with the control groups, BA moieties secondary BAs and glyco conjugated BAs appeared significantly reduced for all three BSH inhibitors treatments and for CAPE and riboflavin treated groups respectively. With RS and NRS stratification these effects were maintained. In addition, TLCA emerged as significantly reduced in RS animals with BSH inhibitor riboflavin.

**Table 2:** Quantity of individual bile acids detected in ileal samples from chickens at end point (aged 28 days) treated or not with BSH inhibitors CAPE, riboflavin and carnosic acid.

	Analyte (ng/mg)	Control	Treatments		
			CAPE	Riboflavin	Carnosic acid
	Taurine	31756.7 ± 21524.1	21578.8 ± 12308.8	17365.8 ± 8604.72	20219.9 ± 13145
1°	CA	18345.9 ± 13958	20972.6 ± 24376.8	20951.9 ± 21507	21497.1 ± 21009.5
	CDCA	87807.5 ± 54654.3	71870.5 ± 73024.2	87413.3 ± 58895.8	81940.2 ± 87493
2° and 3°	DCA	93.62 ± 48.8161	110.46 ± 180.62	67.96 ± 45.2455	80.32 ± 69.0979
	LCA	454.9 ± 139.222	203.43 ± 102.195	128.02 ± 22.5331	223.81 ± 146.359
	UDCA	30 ± 13.9709	20.21 ± 10.4807	23.17 ± 11.3589	28.48 ± 21.5456
Free	DHCA	15.03 ± 11.0942	22.04 ± 11.1483	19.37 ± 10.5136	20.16 ± 12.134
	HCA	128.7 ± 63.8126	207.72 ± 148.058	157.03 ± 120.701	284.59 ± 299.273
	HDCA	110.73 ± 64.4569	81.24 ± 39.973	81.41 ± 49.4506	77.72 ± 45.1353
	7-Keto LCA	792.32 ± 562.116	1208.6 ± 1017.19	928.98 ± 803.207	1724.7 ± 3102.9
	MCA	31.04 ± 16.4731	14.65 ± 6.4469	10.53 ± 4.87751	15.76 ± 11.2074
Tauro-conjugated	TCA	176824 ± 141916	188001 ± 167087	337629 ± 307554	298425 ± 229751
	THCA	909.59 ± 617.726	925.76 ± 635.741	1637.49 ± 1285.57	1833.4 ± 983.69
	TCDCA	393422 ± 313666	261438 ± 230898	462709 ± 293324	420155 ± 229029
	TUDCA	903.58 ± 569.553	628.88 ± 447.07	1343.18 ± 793.58	1028.05 ± 626.043
	TDCA	231.21 ± 141.681	158.14 ± 105.343	301.33 ± 202.909	411.91 ± 454.826
	THDCA	1306.84 ± 826.478	1340.12 ± 987.258	3307.76 ± 2348.69	1658.71 ± 886.923
	TLCA	135.51 ± 59.7155	87.88 ± 70.023	215.82 ± 188.725	174.94 ± 129.549
Glyco-conjugated	GCA	885.3 ± 1054.44	586.53 ± 392.118	1652.2 ± 2115.93	1289.44 ± 1198.7
	GHCA	61.18 ± 30.9591	47.97 ± 13.6454	41.31 ± 16.6029	48.24 ± 18.7021
	GHDCA	127.72 ± 53.0436	88.46 ± 28.5804	73.82 ± 22.4606	92.14 ± 29.7437
	GCDCA	572.46 ± 193.191	327.53 ± 169.58	691.44 ± 412.021	749.93 ± 759.147
	GUDCA	125.04 ± 46.511	80.48 ± 40.9429	55.39 ± 10.6329	95.38 ± 36.2647
	GDCA	125.59 ± 34.2556	78.98 ± 40.8439	46.24 ± 14.8928	89.35 ± 47.7493
	GLCA	14.24 ± 7.45603	9.27 ± 4.95077	6.27 ± 4.07187	10.59 ± 8.0987
Muricholics	α-MCA	1250.77 ± 984.831	2035.6 ± 1539.72	1919.35 ± 1833.79	2408.9 ± 2679.91
	β-MCA	1250.77 ± 984.831	2035.6 ± 1539.72	1919.35 ± 1833.79	2408.9 ± 2679.91
	ω-MCA	233.53 ± 109.009	437.71 ± 320.029	631.33 ± 601.471	612.78 ± 684.997
	α-TMCA	11587 ± 8181.47	10204.6 ± 6397.87	23976.8 ± 17716.8	17951 ± 10976.2
	β-TMCA	11587 ± 8181.47	10204.6 ± 6397.87	23976.8 ± 17716.8	17951 ± 10976.2
	ω-TMCA	11587 ± 8181.47	10204.6 ± 6397.87	23976.8 ± 17716.8	17951 ± 10976.2
Totals	Total BA	695718 ± 423098	559958 ± 423521	945071 ± 617788	851183 ± 508046
	Total 1° BA	107827 ± 60839.3	95559 ± 96976.1	111107 ± 75539.5	106788 ± 109946
	Total 2° BA	659.25 ± 184.045	395.13 ± 234.535	277.39 ± 107.463	381.85 ± 237.343
	Total Free BA	108487 ± 60911.7	95954.1 ± 97140.1	111384 ± 75628.3	107170 ± 110110
	Total Tauro BA	585320 ± 428580	462785 ± 368436	831120 ± 565041	741638 ± 442340
	Total Glyco BA	1911.53 ± 1148	1219.22 ± 501.886	2566.67 ± 2482.77	2375.07 ± 1958.65

Data is shown as mean ± SD. All values are expressed in ng/mg.

**Table 3:** Summary of statistical relevance for individual ileal bile acid levels from chickens at end point (aged 28 days) treated or not with BSH inhibitors.

		All samples			Responders		
		Statistical significance vs control			Statistical significance vs Control		
	Analyte (ng/mg)	CAPE	Riboflavin	Carnosic acid	CAPE	Riboflavin	Carnosic acid
	Taurine	ns	ns	ns	ns	ns	ns
1°	CA	ns	ns	ns	ns	ns	ns
	CDCA	ns	ns	ns	↓ ^*^	ns	ns
2° and 3°	DCA	ns	ns	ns	↓^$^	ns	ns
	LCA	↓^$^	↓^$$$^	↓^$^	↓ ^*^	↓^$^	ns
	UDCA	ns	ns	ns	ns	ns	ns
Free	DHCA	ns	Ns	ns	ns	ns	ns
	HCA	ns	Ns	ns	ns	ns	ns
	HDCA	ns	ns	ns	ns	ns	ns
	7-Keto LCA	ns	ns	ns	ns	ns	ns
	MCA	↓ ^*^	↓^$$^	↓^*^	↓ ^*^	↓ ^*^	ns
Tauro-conjugated	TCA	ns	ns	ns	ns	ns	ns
	THCA	ns	ns	ns	ns	ns	ns
	TCDCA	ns	ns	ns	ns	ns	ns
	TUDCA	ns	ns	ns	ns	ns	ns
	TDCA	ns	ns	ns	ns	ns	ns
	THDCA	ns	ns	ns	ns	ns	ns
	TLCA	ns	ns	ns	↓ ^* *^	ns	ns
Glyco-conjugated	GCA	ns	ns	ns	ns	ns	ns
	GHCA	ns	ns	ns	ns	ns	ns
	GHDCA	ns	↓^$^	ns	ns	↓ ^*^	ns
	GCDCA	↓^**^	ns	ns	ns	ns	ns
	GUDCA	↓^*^	↓^$$^	ns	ns	↓ ^*^	ns
	GDCA	↓^*^	↓^$$$^	ns	ns	↓ ^* *^	ns
	GLCA	ns	↓^$^	ns	ns	↓ ^*^	ns
Muricholics	α-MCA	ns	ns	ns	ns	ns	ns
	β-MCA	ns	ns	ns	ns	ns	ns
	ω-MCA	ns	ns	ns	ns	ns	ns
	α-TMCA	ns	ns	ns	ns	ns	ns
	β-TMCA	ns	ns	ns	ns	ns	ns
	ω-TMCA	ns	ns	ns	ns	ns	ns
Totals	Total BA	ns	ns	ns	ns	ns	ns
	Total 1° BA	ns	ns	ns	↓ ^*^	ns	ns
	Total 2° BA	↓ ^*^	↓^$$^	↓^*^	↓ ^*^	↓^$^	ns
	Total Free BA	ns	ns	ns	ns	ns	ns
	Total Tauro BA	ns	ns	ns	ns	ns	ns
	Total Glyco BA	ns	ns	ns	ns	ns	ns

All values are expressed in ng/mg. Statistical significance was calculated using Kruskal wallis: $p < 0.05; $$p < 0.01; $$$p < 0.001 followed by Mann Whitney t-Test (2-tailed): *p < 0.05; **p < 0.01; ***p < 0.001.

**Figure 3:**
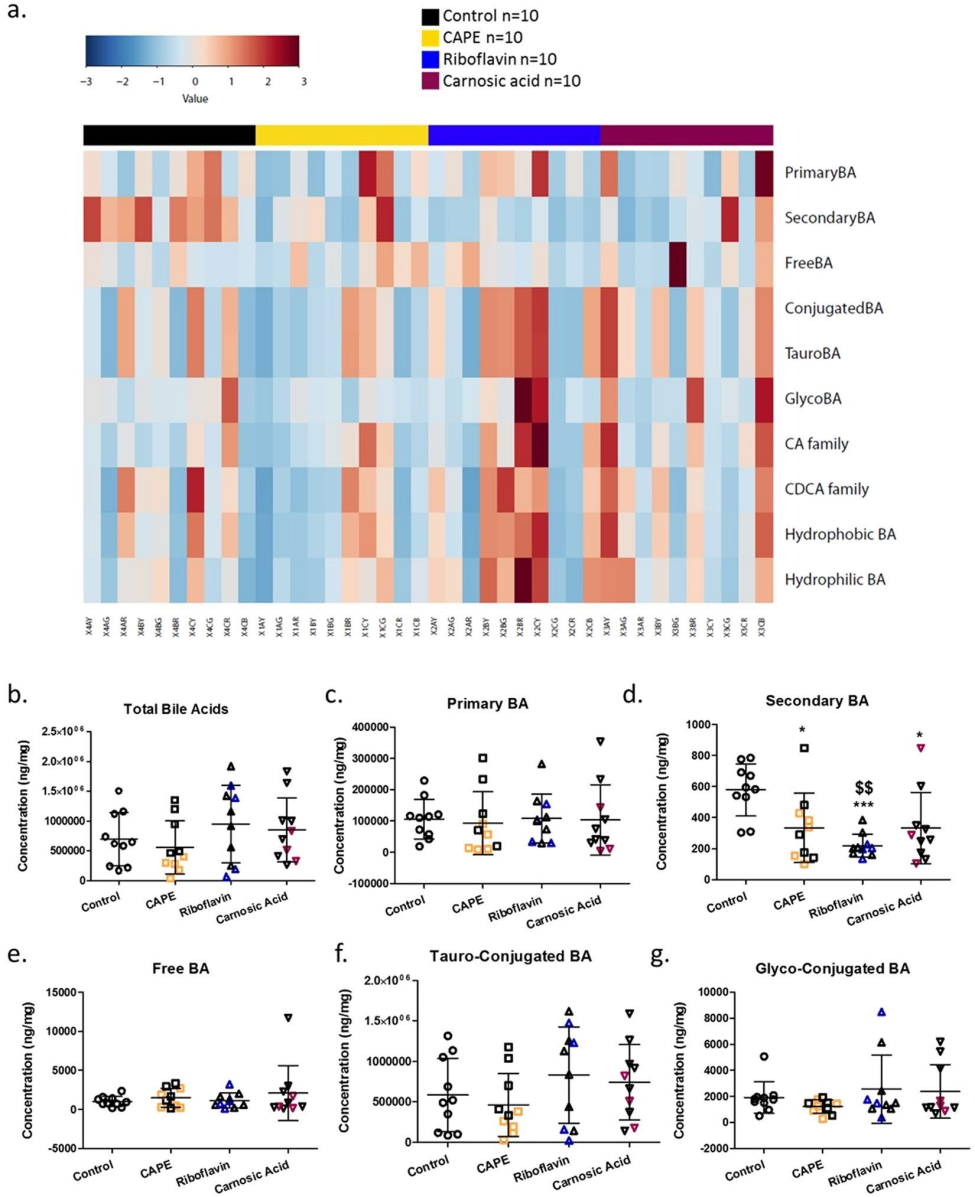
Assessment of the effects of BSH inhibitors on ileal bile acids classifications. (**a**) Heatplot showing relative representation of bile acids according to their classification/family for individual animals following respective treatments. Significantly altered (**b–g**) bile acid family representations. Coloured subjects represent responder (RS) animals with black subjects classified as non-responders (NRS).

**Figure 4:**
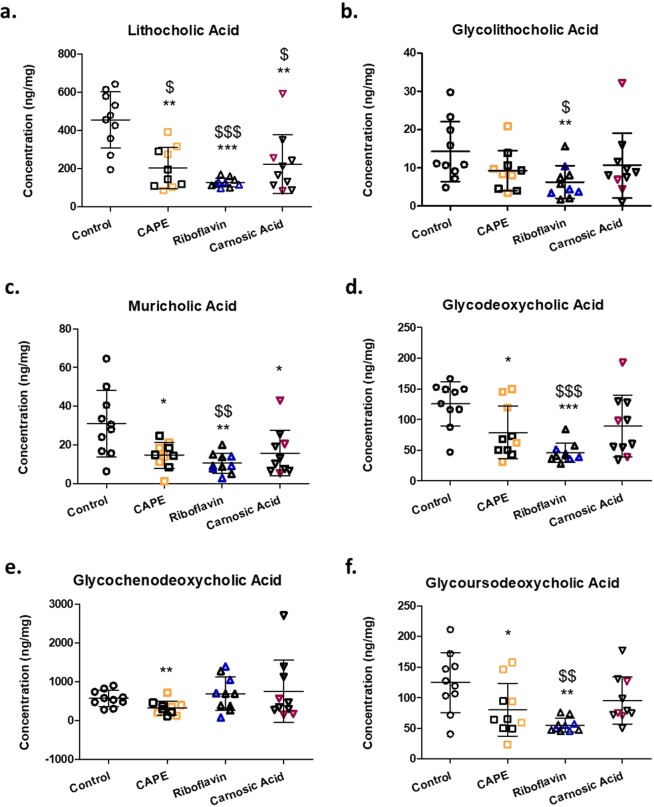
Assessment of the effects of BSH inhibitors on ileal bile acid signatures. Only significantly altered BAs are show (**a**) lithocholic acid, (**b**) glycolithocholic acid, (**c**) muricholic acid, (**d**) glycodeoxycholic acid, (**e**) glycochenodeoxycholic acid and (**f**) glycoursodeoxycholic acid. Data is represented as mean ± SD. Colored subjects represent responder (RS) animals with black subjects classified as non-responders (NRS).

Heatplots showed enrichment for secondary BA in the majority of control animals relative to BSH inhibitor treated animals (Figs. 3 and 4). Furthermore, BSH inhibitor activity enriched for conjugated BA (tauro and glyco) for the majority of treated animals. Further analysis indicated that while there was a trend towards higher levels of total BA on treated with either riboflavin or carnosic acid they did not reach significance (*P* > 0.05) relative to untreated animals (Fig. 3b). These trends were due to increased levels of tauro and glyco conjugated BAs (Fig. 3f,g), consistent with this, significantly lower levels of secondary BAs were detected for the BSH inhibitor treated groups (Fig. 3d). In addition, all of the BSH inhibitor treatment groups tended towards reduction for taurine relative to untreated control animals again suggesting that BSH inhibitors are active *in vivo*. Subsequently, individual secondary BAs were examined, to confirm that oral administration of each BSH inhibitor led to significantly reduced levels of lithocholic acid (LCA) (*P* < 0.05; Fig. 4a). Riboflavin treatment showed the greatest reduction in LCA (Fig. 4a) and it correlated with a trend towards higher levels of taurolithocholic acid (T-LCA) (Table 2). The level of muricholic acid in the intestine was significantly lowered by all of the treatments (Fig. 4c). Significantly lower levels of glycolithocholic acid, glycodeoxycholic acid, and glycoursodeoxycholic acid (Fig. 4b,d,f), all of which are conjugated secondary bile acids, were evident in the riboflavin treated group with similar trends evident where carnosic acid was applied. CAPE treatment alone, significantly reduced levels of glycochenodeoxycholic acid (Fig. 4e). Taken together these data support effective gastrointestinal BSH inhibition in treated animals leading to less microbial deconjugation and dehydroxylation to secondary BAs.

### BSH inhibitor treatment altered circulating levels of bile acid

**Figure 5:**
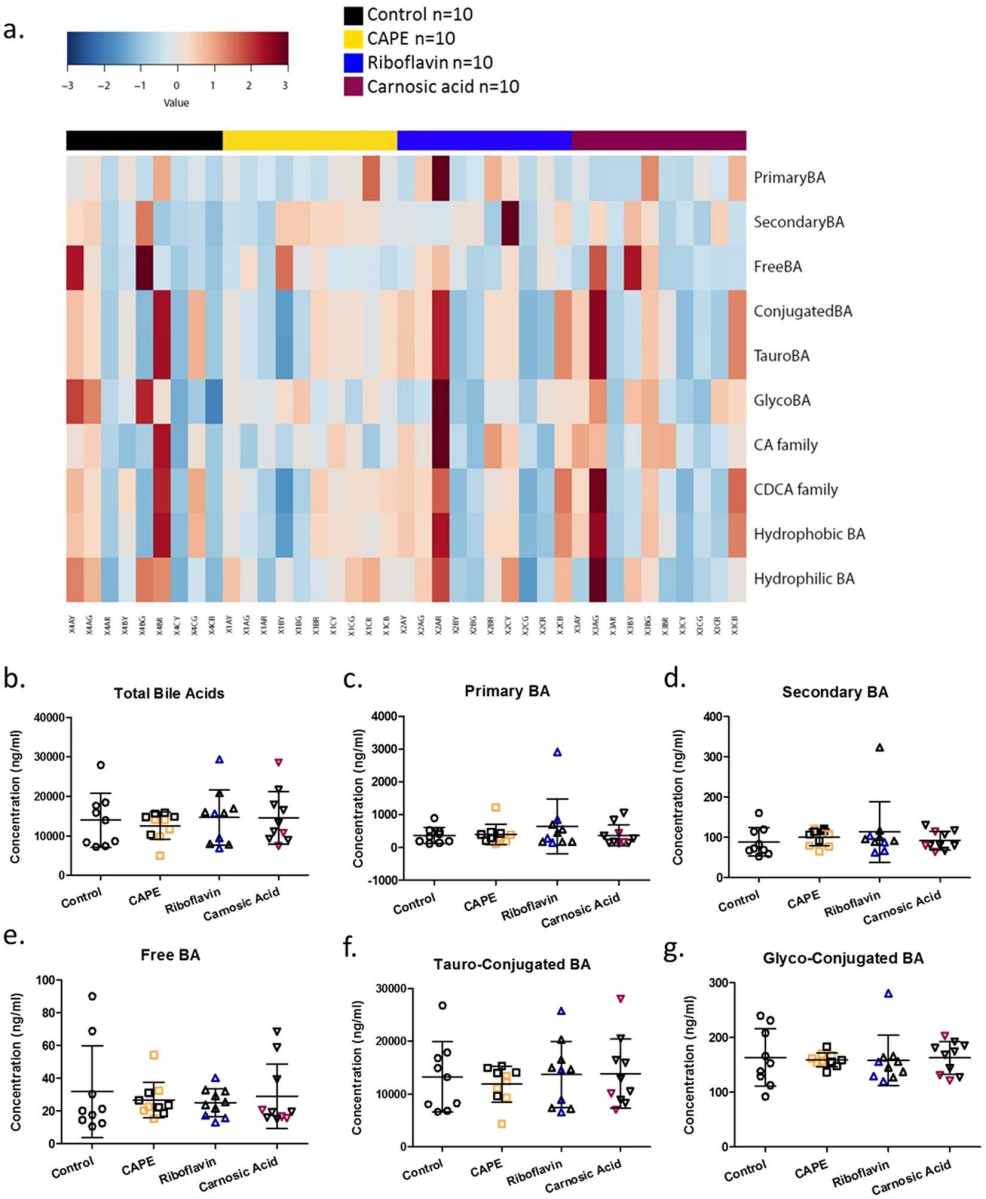
Assessment of the effects of BSH inhibitors on circulating bile acid classifications: (**a**) Heatplot showing relative representation of bile acids according to their classification/family for individual animals following BSH inhibitor treatments. Significantly altered (**b–g**) bile acid family representations. Data is represented as mean ± SD. Coloured subjects represent responder (RS) animals with black subjects classified as non-responders (NRS).

**Figure 6:**
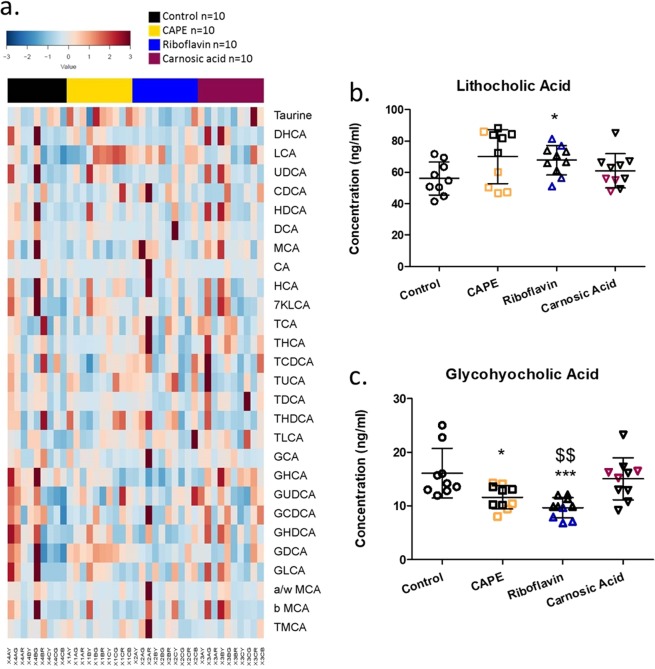
Assessment of the effects of BSH inhibitors on circulating bile acid signatures (**a**) heatplot showing relative representation of individual bile acid moieties for individual animals following their respective treatments. (**b**) lithocholic acid and (**c**) glycohyocholic acid levels are significantly altered in plasma samples following either CAPE or riboflavin treatments, similar trends are evident with carnosic acid. Data is represented as mean ± SD. Coloured subjects represent responder (RS) animals with black subjects classified as non-responders (NRS).

BAs actively transported into enterohepatic circulation are detectible in the blood. Table 4 summarizes the levels of different BA moieties detected in the plasma of untreated and BSH inhibitor treated animals. Only GHCA was significantly reduced in circulation by BSH inhibitors, whether each group was stratified as RS or not (Table 5). Plasma BAs varied among individual animals for all of the treatment groups (Fig. 5a). However, BA categories: total BAs, total conjugated BAs and their subdivided glyco and tauro conjugates, hydrophobic and hydrophilic BAs as well as secondary BAs showed no significant differences or even trends towards differences in their overall content (Fig. 5a–g). Individual BA moieties were next examined (Fig. 6a) confirming that two bile acids LCA (Fig. 6b) and glyco hyocholic acid (G-HCA) (Fig. 6c) were significantly altered in response to CAPE and riboflavin BSH inhibitor. Riboflavin treatment elevated LCA levels significantly (*P* = 0.0220) however trends towards increased LCA were evident for the remaining BSH inhibitors (Fig. 6b). No significant differences were evident for any other BA moieties in circulation.

**Table 4:** Quantity of individual circulating bile acids from chickens at end point (aged 28 days) treated or not with BSH inhibitors.

	Analyte (ng/ml)	Control	Treatments		
			CAPE	Riboflavin	Carnosic acid
	Taurine	5158.07 ± 899.991	6119.81 ± 1686.87	5128.02 ± 1133.08	5486.31 ± 1619.6
1°	CA	48.9056 ± 39.2005	39.9222 ± 15.3336	136.97 ± 252.093	43.905 ± 30.3887
	CDCA	317.872 ± 199.263	360.322 ± 296.289	505.175 ± 543.544	326.415 ± 276.1
2° and 3°	DCA	26.8833 ± 19.2574	24.5611 ± 9.93741	42.06 ± 71.8834	25.795 ± 14.5771
	LCA	56.0667 ± 9.89854	72.6167 ± 15.1554	67.685 ± 8.9165	60.975 ± 10.3039
	UDCA	5.25556 ± 5.5565	4.88889 ± 3.564	3.655 ± 1.58058	4.97 ± 3.98812
Free	DHCA	3.57778 ± 4.48993	2.55 ± 1.80509	1.48 ± 1.06775	3.28 ± 3.97355
	HCA	7.82778 ± 6.25811	7.41111 ± 3.32138	7.135 ± 4.83591	6.9 ± 4.69628
	HDCA	22.5333 ± 21.043	19.0778 ± 12.0967	16.285 ± 8.85362	22.505 ± 18.1956
	7-Keto LCA	7.99444 ± 5.45601	8.65556 ± 2.31078	6.695 ± 2.01339	7.67 ± 4.621
	MCA	5.84444 ± 3.79469	3.94444 ± 1.21528	6.25 ± 3.46143	6.19 ± 2.42
Tauro-conjugated	TCA	1801.21 ± 1397.4	1707.06 ± 582.066	2398.61 ± 1661.79	2185.07 ± 903.695
	THCA	16.2111 ± 5.32326	16.3778 ± 2.41972	19.77 ± 17.0198	21.66 ± 8.78532
	TCDCA	11004.2 ± 4887.34	9535.42 ± 3075.93	10664.2 ± 4217.4	11251 ± 5549.55
	TUDCA	31.0833 ± 8.5127	32.6222 ± 9.67664	34.755 ± 11.8205	31.56 ± 19.5362
	TDCA	13.7944 ± 4.11099	13.7 ± 1.29615	12.97 ± 3.29524	19.305 ± 14.531
	THDCA	70.3333 ± 39.0954	70.9611 ± 31.3462	80.48 ± 34.1904	56.585 ± 27.8863
	TLCA	15.25 ± 7.02416	19.0833 ± 4.97259	25.135 ± 17.9194	21.44 ± 8.82028
Glyco-conjugated	GCA	20.1278 ± 10.5564	17.7667 ± 3.73653	26.605 ± 28.3066	20.32 ± 5.24525
	GHCA	16.1056 ± 4.36447	11.7444 ± 2.16826	9.655 ± 1.78556	15.045 ± 3.70732
	GHDCA	22.1611 ± 6.134	21 ± 3.24953	18.64 ± 3.21044	21.91 ± 4.58633
	GCDCA	50.1278 ± 14.0757	47.9389 ± 7.58708	51 ± 16.2255	52.435 ± 12.1855
	GUDCA	24.5389 ± 8.98062	25.7167 ± 7.4087	24.935 ± 6.17823	24.79 ± 6.87193
	GDCA	26.6111 ± 12.6429	31.7667 ± 2.62245	24.63 ± 3.26567	25.21 ± 5.55944
	GLCA	3.78889 ± 3.28488	3.14444 ± 1.60476	2.9 ± 1.71625	3.205 ± 2.36859
Muricholics	α-MCA	19.4944 ± 14.6791	15.6889 ± 10.5338	38.98 ± 75.1296	18.03 ± 14.1154
	β-MCA	5.83889 ± 6.58765	4.23333 ± 4.04523	5.36 ± 4.4882	6.23 ± 6.33862
	ω-MCA	19.4944 ± 14.6791	15.6889 ± 10.5338	38.98 ± 75.1296	18.03 ± 14.1154
	α-TMCA	299.844 ± 122.856	289.883 ± 103.64	439.265 ± 527.061	260.89 ± 98.2334
	β-TMCA	299.844 ± 122.857	289.883 ± 103.65	439.265 ± 527.062	260.89 ± 98.2335
	ω-TMCA	299.844 ± 122.858	289.883 ± 103.66	439.265 ± 527.063	260.89 ± 98.2336
Totals	Total BA	13931.9 ± 6497.58	12396.9 ± 3408.11	14663.1 ± 6641.96	14532.3 ± 6333.29
	Total 1° BA	411.039 ± 241.842	436.411 ± 312.741	703.525 ± 873.1	412.64 ± 313.966
	Total 2° BA	105.483 ± 48.5805	116.256 ± 24.3109	126.03 ± 78.1179	109.275 ± 35.4238
	Total Free BA	516.522 ± 247.651	552.667 ± 313.649	829.555 ± 869.27	521.915 ± 316.28
	Total Tauro BA	13251.9 ± 6283.48	11685.1 ± 3322.59	13675.2 ± 5964.74	13847.5 ± 6198.93
	Total Glyco BA	163.461 ± 49.4592	159.078 ± 12.6049	158.365 ± 43.5747	162.915 ± 27.9597

Data is shown as mean ± SD. All values are expressed in ng/ml.

**Table 5:** Summary of statistical relevance for individual circulating bile acid levels from chickens at end point (aged 28 days) treated or not with BSH inhibitors.

		All animals			Responders		
		Statistical significance vs Control			Statistical significance vs Control		
	Analyte (ng/ml)	CAPE	Riboflavin	Carnosic acid	CAPE	Riboflavin	Carnosic acid
	Taurine	ns	ns	ns	ns	ns	ns
1°	CA	ns	ns	ns	ns	ns	ns
	CDCA	ns	ns	ns	ns	ns	ns
2° and 3°	DCA	ns	ns	ns	ns	ns	ns
	LCA	ns	ns	ns	ns	↑^*^	ns
	UDCA	ns	ns	ns	ns	ns	ns
Free	DHCA	ns	ns	ns	ns	ns	ns
	HCA	ns	ns	ns	ns	ns	ns
	HDCA	ns	ns	ns	ns	ns	ns
	7-Keto LCA	ns	ns	ns	ns	ns	ns
	MCA	ns	ns	ns	ns	ns	ns
Tauro-conjugated	TCA	ns	ns	ns	ns	ns	ns
	THCA	ns	ns	ns	ns	ns	ns
	TCDCA	ns	ns	ns	ns	ns	ns
	TUDCA	ns	ns	ns	ns	ns	ns
	TDCA	ns	ns	ns	ns	ns	ns
	THDCA	ns	ns	ns	ns	ns	ns
	TLCA	ns	ns	ns	ns	ns	ns
Glyco-conjugated	GCA	ns	ns	ns	ns	ns	ns
	GHCA	↓^*^	↓^$$^	ns	ns	↓$	ns
	GHDCA	ns	ns	ns	ns	ns	ns
	GCDCA	ns	ns	ns	ns	ns	ns
	GUDCA	ns	ns	ns	ns	ns	ns
	GDCA	ns	ns	ns	ns	ns	ns
	GLCA	ns	ns	ns	ns	ns	ns
Muricholics	α-MCA	ns	ns	ns	ns	ns	ns
	β-MCA	ns	ns	ns	ns	ns	ns
	ω-MCA	ns	ns	ns	ns	ns	ns
	α-TMCA	ns	ns	ns	ns	ns	ns
	β-TMCA	ns	ns	ns	ns	ns	ns
	ω-TMCA	ns	ns	ns	ns	ns	ns
Totals	Total BA	ns	ns	ns	ns	ns	ns
	Total 1° BA	ns	ns	ns	ns	ns	ns
	Total 2° BA	ns	ns	ns	ns	ns	ns
	Total Free BA	ns	ns	ns	ns	ns	ns
	Total Tauro BA	ns	ns	ns	ns	ns	ns
	Total Glyco BA	ns	ns	ns	ns	ns	ns

All values are expressed in ng/ml. Statistical significance was calculated using Kruskal wallis: $p < 0.05; $$p < 0.01; $$$p < 0.001 followed by Mann Whitney t-Test (2-tailed): *p < 0.05; **p < 0.01; ***p < 0.001.

### Assessment of BA content as receptor agonists and antagonists

**Figure 7:**
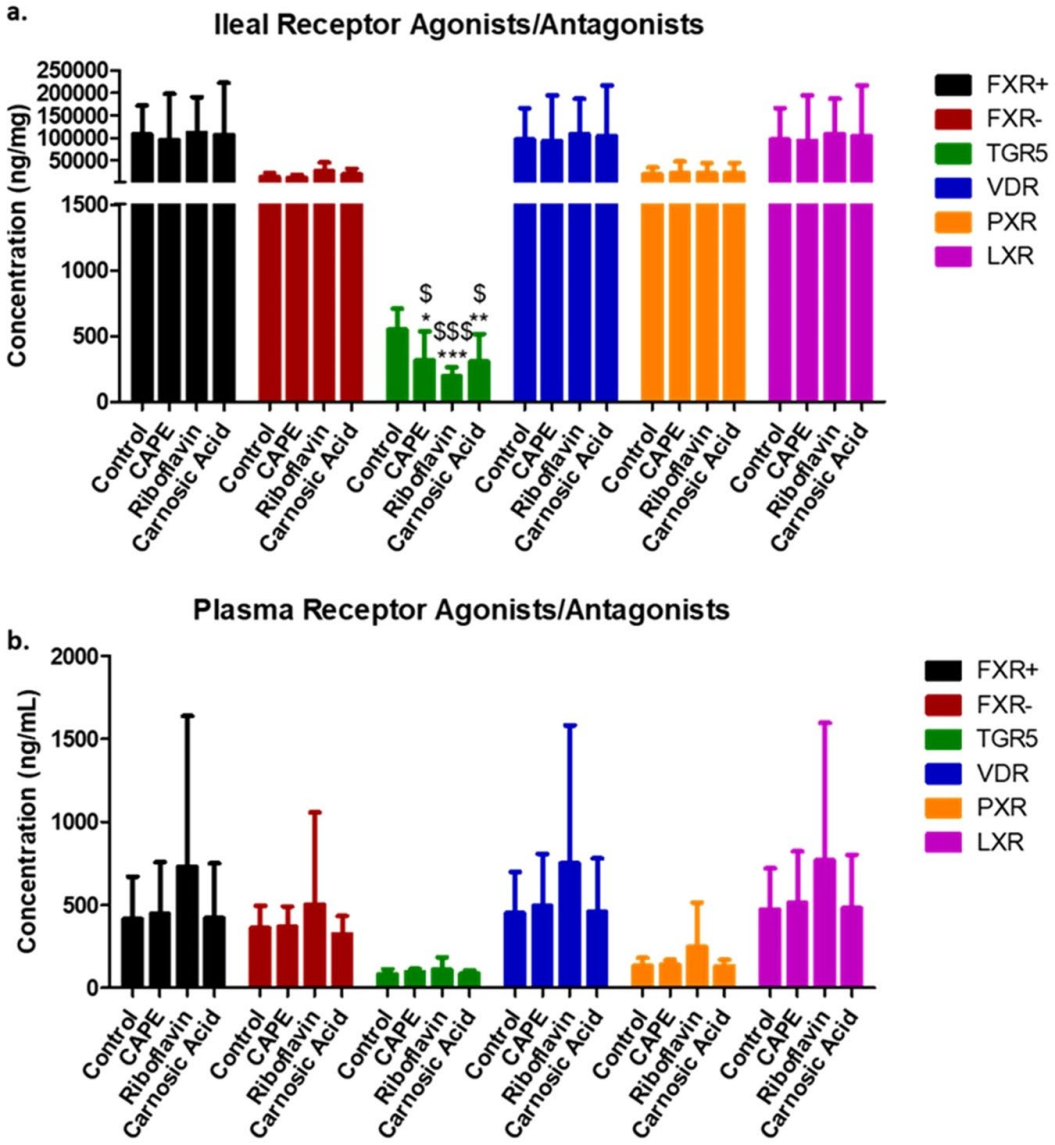
Prediction of (**a**) Ileal and (**b**) circulating bile acid receptor activation post BSH inhibitor treatment relative to non treated animals. Agonists (+) and antagonists (−) for FXR; agonists for TGR5, VDR and PXR are represented. Data is represented as mean ± SD.

BA moieties can differentially alter gene expression through their interactions with bile acid receptors (BARS) including farnesoid X receptor (FXR), Takeda G protein couple receptor (TGR5), Vitamin D receptor (VDR) Pregnane X receptor (PXR) and Liver X receptor (LXR). The BA signatures generated could predict potential receptor interaction and signaling both locally in the ileum and in circulation to other tissues (Fig. 7). In the ileal, the combined levels of FXR antagonist (UDCA, TUDCA, GUDCA, T-MCA) was similar between the different groups. Interestingly, for all groups, the concentration FXR agonist (CA, CDCA, DCA, α-MCA and β- MCA) were within the same range but the level exceeded antagonist concentrations *ca*. 100 fold. In contrast, TGR5 agonists (LCA, DCA) were significantly reduced for all BSH inhibitor treatments compared to control animals. The levels of BA moieties ligands for VDR (LCA, CDCA, DCA, CA), PXR (LCA, DCA, CA) and LXR (CA, LCA, DCA, CDCA, HDCA) remained unchanged (Fig. 7). BAs in circulation are relatively low compared to ileal BA levels (Fig. 7b), here no significant differences were detected between the treatment groups on the basis of NR or GPCR agonism. Taken together, this work indicates that signals that may be elicited at the level of the gut do not translate systemically.

### BSH inhibitor treatment significantly altered host transcriptome profiles in the liver and the ileum

In order to assess gene transcription changes in the gut and in the liver one BSH inhibitor, carnosic acid (n = 4) was compared relative to untreated animals (n = 4), in order to assess gene transcription changes in the gut and in the liver by RNA-Seq. For BSH inhibitor treatment assessment they represented 1 RS and 3 NRS, based on body weight as described above. The high quality RNAs (reflected by RIN, Table S1) were extracted from tissue samples and applied for mRNA-Seq library construction and sequencing (Illumina Hi-Seq platform (150-cycle paired end). More than 685 million raw reads in pairs were obtained they ranged from 9.2 to 162.7 M raw reads per sample (Table S1). The mapping rate of RNA samples from liver samples and ileum samples ranged from 61.2% to 88.2%.

In response to carnosic acid, 607 DEGs (495 up-regulated and 112 down-regulated) were evident from liver tissue analysis while 215 DEGs (116 up-regulated and 99 down-regulated) were detected in the ileum. As expected, many DEGs are consistent with the reported function of carnosic acid, as an antioxidant and anti-inflammatory agent, partly demonstrating the success of RNA-Seq analysis. For example, of more than 600 liver DEGs, approximately 80 DEGs were directly related to immunity and inflammatory response (data not shown). A notable panel of DEGs involved in bile biosynthesis, lipid metabolism and relevant physiology were also observed. The representative DEGs in response to carnosic acid treatment are described in Table 6.

**Table 6:** Differentially expressed genes (DEGs) in response to carnosic acid treatment in liver and ileum tissues.

Gene^a^	Description of gene product	Fold change by RNA-Seq	Fold change by qRT-PCR^b^
**Liver**			
Upregulated			
00000007416 (*CD3E*)	CD3e molecule	1.78	ND
00000013723 (*OASL*)	Oligoadenylate synthetase-like	10.39	ND
00000000141 (*BLB1*)	Major histocompatibility complex	5.33	ND
00000012834 (*AKR1D1*)	Aldo-keto reductase	2.70	3.02
00000002988 (*PHGDH*)	Phosphoglycerate dehydrogenase	2.13	ND
00000006995 (*APOD*)	Apolipoprotein D	4.40	ND
00000015662 (*LIPI*)	Lipase	2.03	ND
00000029150 (*AK1*)	Adenylate kinase	2.74	ND
00000011360 (*PRKAG3*)	Protein kinase	3.89	ND
00000005795 (*CYP2C23a*)	Cytochrome P450 family	4.72	ND
00000004436 (*CYP3A80*)	Cytochrome P450 family	1.76	1.66
00000010469 (*CYP4B7*)	Cytochrome P450 family	2.13	ND
00000008518 (*LOC417253*)	Glutamine synthetase-like	2.02	ND
00000016325 (*GSTA3*)	Glutathione S-transferase	3.85	2.19
00000008409 (*GSTO1*)	Glutathione S-transferase	1.79	ND
00000028551 (*LOC100859645*)	Glutathione S-transferase-like	2.34	ND
00000011468 (*IGFBP5*)	Growth factor binding protein	3.88	ND
**Downregulated**			
00000024272 (*S100A9*)	Calcium binding protein	−4.02	−3.98
00000003876 (*TIMD4*)	T-cell immunoglobulin and mucin	−1.76	ND
00000022815 (*AvBD1*)	Avian beta-defensin 1	−22.37	ND
00000016669 (*AvBD2*)	Avian beta-defensin 2	−83.19	ND
00000016668 (*AvBD6*)	Avian beta-defensin 6	−39.80	−15.50
00000027973 (*CATH1*)	Cathelicidin-1	−24.74	ND
00000019696 (*CATH2*)	Cathelicidin-2	−11.43	ND
00000016710 (*CYP39A1*)	Cytochrome P450 family	−1.63	ND
00000016426 (*MAP3K15*)	Mitogen-activated protein kinase	−2.86	ND
00000021039 (*HKDC1*)	Hexokinase	−2.87	ND
00000005845 (*SLC7A5*)	Solute carrier family	−1.99	ND
**Ileum**			
Upregulated			
00000013723 (*OASL*)	2′-5′-oligoadenylate synthetase-like	3.35	ND
00000027247 (*EOMES*)	Eomesodermin	3.18	ND
00000016400 (*RSAD2*)	Radical S-adenosyl methionine domain	2.81	ND
00000007395 (*ABCC2*)	ATP-binding cassette	2.18	ND
00000013969 (*ALDH8A1*)	Aldehyde dehydrogenase family	1.7	ND
00000016325 (*GSTA3*)	Glutathione S-transferase	2.44	2.22
00000015702 (*PTGR1*)	Prostaglandin reductase	2.45	ND
00000023933 (*G0S2*)	G0/G1 switch	4.17	3.10
00000008845 (*HAO1*)	Hydroxyacid oxidase	2.35	ND
00000002466 (*SLC2A5*)	Solute carrier family	1.92	ND
00000013969 (*ALDH8A1*)	Aldehyde dehydrogenase	1.70	ND
00000005795 (*CYP2C23a*)	Cytochrome P450 family	2.31	ND
00000011219 (*GCGR*)	Glucagon receptor	2.85	ND
**Downregulated**			
00000016164 (*ABCG1*)	ATP-binding cassette	−3.71	−2.60
00000015081 (*ACER2*)	Alkaline ceramidase	−3.20	−1.35
00000028519 (*CCR10*)	C-C motif chemokine receptor	−3.61	−2.33
00000015441 (*CD247*)	CD247 molecule	−1.77	ND
00000006480 (*TCF7*)	Ttranscription factor	−2.92	ND
00000006707 (*NOX1*)	NADPH oxidase	−2.60	ND
00000005572 (*NOXO1*)	NADPH oxidase organizer	−3.32	ND
00000001857 (*FAM132A*)	Family with sequence similarity 132 member	−2.35	ND
00000014766 (*HAO2*)	Hydroxyacid oxidase	−3.78	ND
00000026817 (*LOC771638*)	Hydrogenase/reductase	−2.89	ND

Results were determined based on DAVID bioinformatics resources (https://david.ncifcrf.gov/). The genes denoted as Upregulated are those having significantly higher expression level in carnosic acid treatment group than those in the control group; genes denoted as Downregulated are those having significantly lower expression in carnosic acid treatment group than those in the control group.

^a^Gene is expressed as ENSGALG gene ID number followed by corresponding gene name in parentheses.

^b^Means of four individual tissue samples with triplicate measurement for each sample. ND, not determined.

In addition to those involved genes in immune response (CD3E, OASL, BLB1), antioxidant and anti-inflammatory roles (glutamine synthetase, glutathione S-transferase), highly upregulated genes in liver were associated with BA metabolism (AKR1D1, PHGDH) and secondary metabolites biosynthesis (P450 CYP2C23a, CYP3A80, CYP4B7), lipid metabolism (APOD, LIPI), energy metabolism (AK1), glucose metabolism (PRKAG3), and insulin regulation (IGFBP5) (Table 6). Interestingly, the highest fold increases in liver gene transcription were detected for oligoadenylate synthetase (>10 fold). Oligoadenylate synthase (OAS) proteins sense exogenous nucleic acid and initiate antiviral pathways and for major histocompatibility complex (>5 fold) initiating acquired immune system recognition. Growth factor binding protein (>3.8 fold increased) is a carrier of insulin–like growth factor 1 (IGF-1); IGF-1 can exert positive or negative effects, depending on its levels and distribution to include apoptosis induction, or alteratively, cancerogenic cell proliferation. Negative DEGs in the liver include avium defensins 2, 6 and 1, their expression was reduced 83, 39 and 22 fold respectively. Furthermore, lysosomal innate immunity molecule cathelicidin was reduced 24 fold by carnosic acid treatment.

Ileal DEGs were not as profoundly altered when compared to those of the liver and changes were in the region of 4–5 fold were recorded. Here, upregulation of antioxidant and anti-inflammatory coding genes and transcription factors were evident, including oligoadenylate synthetase (5.5 fold) eomesodermin (3.1 fold) and glutathione S transferase (2.44 fold). Interestingly cell division checkpoint G0/G1 switch was up regulated (4.17 fold). Regarding glucose metabolism, the fasting and energy reserve metabolism activator glucagon receptor was increased (2.8 fold).

Confirmation and validation of RNA-Seq analysis was performed by examining a number of DEGs associated with BA and lipid metabolism in the liver or ileum, by qRT-PCR analysis (Table 6). The qRT-PCR results are consistent with RNA-Seq analysis (Table 6), validating the expression of these genes and further demonstrating the integrity of RNA-Seq analysis.

### GO enrichment analysis of differentially expressed genes

**Figure 8:**
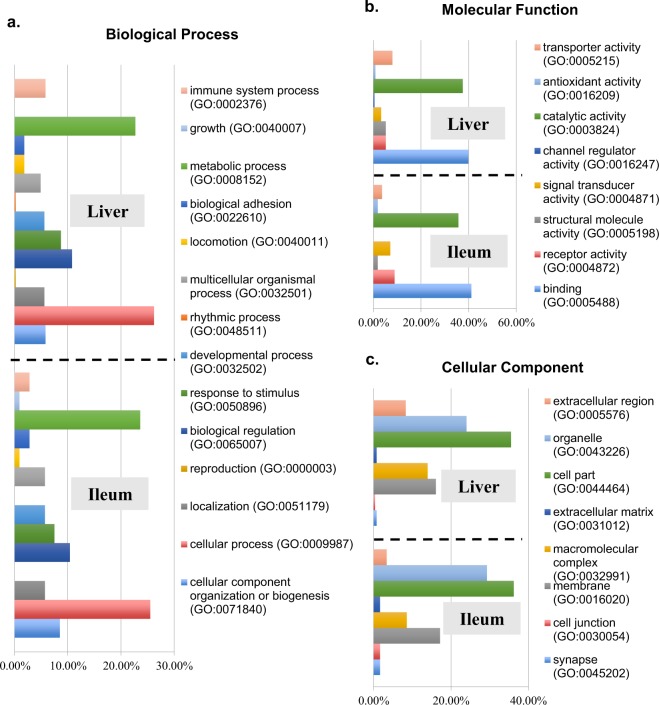
Functional enrichment of Gene Ontology or up-regulated transcripts detected from chicken liver and ileum in response to carnosic acid treatment. Functional enrichment of differentially abundant genes was analyzed by Gene Ontology Consortium with GO Enrichment Analysis tool (http://www.geneontology.org/page/go-enrichment-analysis). The set of differentially abundant genes was functionally annotated with DAVID bioinformatics resources (https://david.ncifcrf.gov/). (**a**) Up-regulated genes classified with the specific biological process terms, (**b**) Up-regulated genes classified with the specific molecular function terms, (**c**) Up-regulated genes classified with the specific cellular component terms.

**Figure 9:**
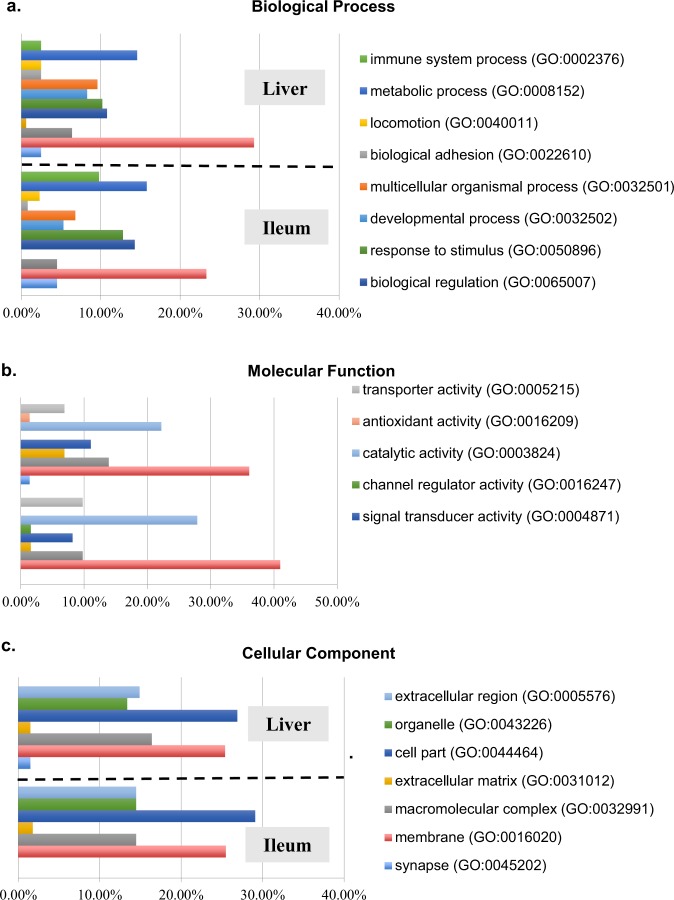
Functional enrichment of GO terms of down-regulated transcripts in chicken liver and ileum in response to carnosic acid treatment. Functional enrichment of differentially abundant genes was analyzed by Gene Ontology Consortium with GO Enrichment Analysis tool (http://www.geneontology.org/page/go-enrichment-analysis). The set of differentially abundant genes was functionally annotated with DAVID bioinformatics resources (https://david.ncifcrf.gov/). (**a**) Down-regulated genes classified with the specific biological process terms, **(b**) Down-regulated genes classified with the specific molecular function terms, (**c**) Down-regulated genes classified with the specific cellular component terms.

In order to compare functional enrichment between the carnosic acid and non-treated groups a total of 607 DEGs (495 up-regulated and 112 down-regulated) from liver samples and 215 DEGs (116 up-regulated and 99 down-regulated) from the ileum were subsequently applied for GO term enrichment analysis (Figs. 8 and 9). The majority of alterations were associated with metabolic processes with 22.7% and 23.6% of up-regulated genes in liver and ileum, respectively and 14.6% and 15.8% down-regulated genes in liver and ileum, respectively. This was followed by cellular processes with 26.2% and 25.5% up-regulated genes in liver and ileum, respectively and 29.3% and 23.3% down-regulated genes in liver and ileum, respectively and then biological regulation with10.8% and 10.4% up-regulated genes in liver and ileum, respectively and 10.8% and 14.3% down-regulated genes in liver and ileum, respectively (Figs. 8 and 9).

### Biochemical analyses of serum samples

The effects of all three BSH inhibitors relative to non treated controls were assessed for metabolic indicators including circulating lipid associated parameters (cholesterol, triglycerides, VLDL) and for glucose (Table 7). While BSH inhibitor treatment did not induce significant changes in the levels of circulating glucose (*P* = 0.08) glucose levels tended to be lower for all three BSH inhibitor treated groups. In contrast, total cholesterol levels appeared elevated in the treated groups (*P* = 0.33) a trend that was also consistent for VLDL (*P* = 0.18). Circulating triglyceride levels remained normal and equivalent among these different groups.

**Table 7:** Biochemical analyses of chicken serum following BSH inhibitor treatment.

Biochemical Parameters	Control	CAPE	Riboflavin	Carnosic Acid	*P* value
Glucose (mg/dL)	224.49 ± 12.78	189.96 ± 7.98	186.32 ± 6.81	186.58 ± 7.45	0.08
Total Cholesterol (mg/dL)	96.94 ± 6.98	91.84 ± 6.61	105.26 ± 7.57	110.61 ± 7.96	0.33
Triglycerides (mg/dL)	42.69 ± 3.58	40.20 ± 3.35	39.35 ± 3.28	41.86 ± 3.47	0.85
VLDL (mmol/L)	11.53 ± 0.67	14.35 ± 1.49	16.43 ± 2.55	14.38 ± 1.95	0.18

The concentrations of serum glucose, triglycerides, total cholesterol and very low density lipoprotein (VLDL) were expressed as mean ± standard error. Statistical significant level of difference is defined by P < 0.05.

## Discussion

The widespread use of AGPs are no longer encouraged through the food chain so that alternative approaches to mimic the effects of AGPs are being explored. AGP is correlated with reduced microbial population diversity and is therefore associated with the loss of microbial functionality to induce increased mass in animals for food supply. One such microbial function is BSH enzyme activity, a gateway activity for microbial conversion of BAs so that a palate of BAs are generated with different signaling abilities and lipid solubilization abilities. Increasing evidence implicates BSH activity in modulating host lipid metabolism and energy harvest *in situ*, we therefore speculated that BSH inhibitors could provide promising alternatives to AGPs in order to improve feed efficiency and body weight gain of animals as food.

To test this, in addition to *in vitro* identification of promising BSH inhibitors, examination of the *in vivo* efficacy of specific BSH inhibitor was performed with three novel and promising BSH inhibitors which we have identified and characterized namely riboflavin, CAPE, and carnosic acid^10^. Riboflavin is a vitamin participating in a range of redox reactions in the host^12,13^. It is applied as feed additive at low trace level in poultry feed (only 2.5 ppm) to prevent and control the hypovitaminosis B2. However, long-term dietary supplementation of higher levels of riboflavin as BSH inhibitor for growth promotion in chicken and other food animals has never been explored. In fact, a previous study indicated that dietary supplementation of riboflavin increased feed efficiency and BW gain in pigs^14^, which may also be mediated through inhibition of intestinal BSH activity. Both CAPE and carnosic acid are emerging natural food additives that recently have attracted extensive attention for human and animal application. CAPE and carnosic acid have shown multiple bioactivities, such as anti-carcinogenic, anti-oxidation, and anti-inflammatory^15–19^. However, to date, little information exists concerning the effect of CAPE and carnosic acid on host lipid metabolism and/or growth performance. One study reported that carnosic acid is involved in glucose and lipid metabolism^20^, which is likely partly dependent on the inhibitory effect of carnosic acid on BSH enzyme.

In this study, to better control BSH inhibitor dosage and delivery, we chose to perform cage trial and administered BSH inhibitor to each chick via oral gavage. Metabolomic analysis indicated that dietary supplementation of the BSH inhibitors did inhibit intestinal BSH activity and significantly changed BA profile, particularly in ileal towards higher levels of BA conjugates and lower levels of downstream secondary BAs. This effect can only be due to inhibition of deconjugation to provide BA precursors of secondary BAs^6,7,21,22^. Notably, secondary BA lithocholic acid (LCA), significantly decreased with all BSH inhibitor treatment. It is regarded as a cytotoxic component in animal models, leading to reduced BW gain^23–27^ and it is implicated in colon carcinogenesis^24^. Indeed, addition of LCA to chicken feed increased animal plasma lipid levels, liver size but decreased liver fat deposition while inducing biliary hyperplasia in chicks^26,27^. More recently, LCA was also shown to impair nutrient uptake in rat studies^28^. Importantly, LCA is a ligand for other lipid metabolism signaling factors including such as LXR^29^ and PXR^30^. Its cognate tauro conjugate is decreased in these BSH inhibitor treated groups, which serves as an activator of inflammatory dampening through Takeda Receptor (TGR5) present in a range of tissues including macrophage^31^. TGR5 can also influence host energetics through thyroid hormone activation and increased energy expenditure, browning of white adipose tissue and through FGF21 activation and by stimulating secretion of incretin glucagon-like peptide (GLP)-1, to release insulin to alter blood glucose levels, gastrointestinal motility and satiety. Finally, some BSH inhibitor-mediated BA changes may further enhance host energy harvest, βMCA and TCA, (both FXR activators) were elevated in BSH treatment groups they may alter BA, lipid and glucose metabolism^32,33^.

Despite the evidence to support effective BSH inhibitor treatment in this cage trial, it is not surprising that the differences in BW gain and feed efficiency were not always statistically significant at the different time points examined. BW gain and feed efficiency assessment for broiler chicken are usually large (n = 10 bird per pen with 10 pens per treatment)^11^. BW gain and feed efficiency in this cage trial were lower than those observed in traditional pen trial, since cage design (away from ground with hanging feeder) may have caused loss of feed, reduced accessibility of small chicks to feeder and water, lowered the density of young chicks (3–4 per cage). Consequently, low feed efficiency was observed across all groups. Despite these limitations, this cage trial indicates that all three BSH inhibitors enhanced BW gain and feed efficiency, and also significantly changed the bile acid profile due to inhibition of BSH activity, providing a strong rationale for us to perform large scale pen trials in the future.

In addition to analysis of BA alterations we performed RNA-Seq analysis to evaluate the local and systemic impacts of the BSH inhibitor carnosic acid. The findings from RNA-Seq analysis in this study not only supported *in vivo* inhibition of intestinal BSH by carnosic acid but also showed the influence of this BSH inhibitor upon local (gut) and systemic (liver) regulators of lipid metabolism. In the liver *AKR1D1* was induced by carnosic acid, Tand it functions in BA synthesis to transform 7-α-hydroxycholesterol to primary bile acids (cholic acid and chenodeoxycholic acid)^34^. Notably, expression of a panel of host Cytochrome P450 enzymes, such as *CYP2C23a, CYP3A80, CYP4B7*, are also significantly induced upon carnosic acid treatment (Table 4). P450 enzymes are active determining the fate of cholesterol to either hormones or BAs^35^. To date, the functions of the P450 enzymes are still largely unknown in chicken. Due to the high similarity between chicken CYP3A80 and human CYP3A4 (59% sequence identity), CYP3A80 may catalyze the 6-hydroxylation of LCA as observed for CYP3A4^36,37^.

Of the DEGs identified in ileum tissue samples, the upregulated *G0S2* and the downregulated *ACER2* are of particular interest. The *G0S2* gene is highly conserved in vertebrates and is a master regulator of tissue-specific balance of triglyceride storage and mobilization, partitioning of metabolic fuels between the adipose tissue, the liver and the whole body adaptive energy response^38^. G0S2 is a lipolysis inhibitor by inhibiting adipose triglyceride lipase which is responsible for catalyzing the first step of three stepwise reactions that define lipolysis^38,39^. In addition, *G0S2* is also a target gene for peroxisome proliferator activated receptor (PPARα), a transcription factor participating in lipid metabolism and adipogenesis^40^. ACER2 is critical in metabolism of bioactive lipid sphingolipids^41–43^, downregulation of ACER2 upon carnosic acid may lead to changes in serum LDL, HDL and cholesterol level^44^.

Clearly, all the three selected BSH inhibitors are predicted to display off target effects and our RNA-Seq analysis supports this notion. Carnosic acid is a known anti-oxidant with anti-inflammatory and anti-carcinogenic properties^17,45,46^, and it enhanced a range of immune and inflammation responses in both liver and ileum tissues. Carnosic acid can exert its anti-oxidant effects by the activation of the PI3K/Akt/Nrf2 signaling pathway^17^ to induce the Nrf2-targeted gene *GSTA3*^47,48^, which is also upregulated in ileum in this study. Carnosic acid treatment resulted in liver downregulated endogenous host defense peptide (HDP), including AvBD1, AvBD2, AvBD6, Cathelicidin-1, and Cathelicidin-2, important first line of defense against microbial infections^49–51^ Therefore, further examination of the influence of carnosic acid on susceptibility to microbial infection is highly warranted. Nevertheless, the implications from this and other animal work is that these BSH inhibiting compounds may induce healthy weight gain rather than simply fat mass, therefore the use of these inhibitors could be highly desirable as an alternative to AGPs.

The majority of our knowledge base for food, microbes and BA metabolism comes from rodents and their translation to humans, there are considerable knowledge gaps in animal husbandry^52,53^. There are many incidences where this work does not translate, due simply to copy number classes of enzymes, fundamental metabolic differences and to signaling. The extent to which chicken systems are characterized in terms of metabolism, microbiota, receptor signaling and the host microbe dialogue is an emergent area for discovery particularly in addressing AGP alternatives. Further comprehensive nutritional, physiological, microbiome, genomics and metabolomics measurements associated with BSH inhibitor use are warranted to accurately determine their mode of action, degree of penetrance and indeed whether any growth promotion in animal husbandry can be achieved through healthy means.

## Materials and methods

### Chicken management, experimental design and sample collection

All of the methods described below were performed in accordance with the relevant guidelines and regulations. Chicken experiment was reviewed and approved by the University of Tennessee Institutional Animal Care and Use Committee (IACUC No. 2187). Forty newly hatched male broilers from Hubbard LLC (Pikeville, TN, USA) were raised in the Joseph E. Johnson Animal Research and Teaching Unit (JARTU, Knoxville, TN, USA). 7 days old chicks were randomly allocated into four groups (n = 10/group). They received one of caffeic acid phenethylester (Wuhan Yuancheng Gongchuang Tech, Wuhan, Hubei, China), riboflavin (Bulk Supplements, Henderson, NV, USA), carnosic acid (Purify, Chengdu, Sichuan, China) at 25 mg/kg, or simply control solution (50% propylene glycol), via oral gavage once a day for 21 consecutive days. The dosage was adjusted accordingly based on average chicken BW determined on Day 7, 10, 14, 17, 21, and 24. Water and feed were available *ad libitum* during the project. On Day 7, 10, 14, 17, 21, 24, and 28, BW and feed consumption were recorded. On Day 28, blood samples were collected from the brachial vein of each bird. Following blood sampling, all birds were euthanized by CO_2_ asphyxiation and dissected for sampling. The ileal contents were collected from all birds, transferred to sterile cryogenic vials, immediately frozen on dry ice and stored at −80°C prior to shipment for metabolomic analysis. Approximately 5 cm of ileum from each chicken was collected and rinsed with saline (0.9% sodium chloride, w/v); the cleaned ileum and a sample of liver tissue from each bird were immediately frozen in liquid nitrogen and stored in cryogenic vials at −80°C prior to extraction for RNA-Seq analysis. The blood samples were centrifuged at 4,000 *g* for 15 min; subsequently, the supernatant (serum) was aliquoted in sterile cryogenic vials and stored in −80 °C freezer prior to further analysis.

### Serum biomarker analyses

The concentrations of glucose, triglycerides, total cholesterol, and very low-density lipoprotein (VLDL) in each serum sample were determined by using following commercial kits: Glucose Colorimetric Assay Kit and Triglyceride Colorimetric Assay Kit (Cayman Chemical, Ann Arbor, MI, USA; for glucose and triglyceride), EnzyChrom Cholesterol Assay Kit (Bioassay System, Hayward, CA, USA; for total cholesterol), Chicken very low density lipoprotein, VLDL ELISA kit (Novatein Biosciences, Woburn, MA, USA; for VLDL).

### Bile acid profile analyses

Serum and ileal contents samples from individual chickens were packaged on dry ice and shipped for ‘blinded’ metabolomics measurements and statistical analysis. The extracted BAs from each sample were analyzed by Ultra Performance Liquid Chromatography-Tandem Mass Spectrometry (UPLC-TMS). The procedures for BA extraction, characterization and quantification were detailed in a previous publication^8^.

### RNA extraction and RNA-Sequencing

For each liver or ileum tissue sample (n = 4 for control and for treated groups for each tissue type) total RNA was extracted using Qiagen RNeasy mini kit (Qiagen Inc., Valencia, CA, USA) following the manufacturer’s instructions. The RNA integrity number (RIN), concentration and the 28 S/18 S ratio of RNA were then evaluated using Agilent RNA 600 Nano kit with Agilent 2100 Bioanalyzer (genomics Hub, University of Tennessee Institute of Agriculture, USA). The total RNA samples were stored at −80 °C prior to analysis. Extracted RNA was packaged on dry ice and shipped to the DNA Facility at Iowa State University (Ames, IA, USA) for mRNA-Seq library construction and RNA-Seq. Overall, sixteen sequencing libraries were constructed from RNA samples and subjected to sequencing using Illumina HiSeq 3000 system (150-cycle paired-end high-output sequencing).

### RNA-Seq differential gene expression analysis

The RNA-Seq differential gene expression analysis was performed in the Office of Advanced Research Computing (Rutgers University, NJ, USA). The raw reads were quality trimmed using Trimmomatic-0.33 with leading and trailing Q score 25, minimum length 25 bp, and minimum length 50 bp. The cleaned reads were mapped to *Gallus_gallus* genome - 5.0 version, using Tophat v.2.0.13. The reference genome sequence and annotation files were downloaded from ENSEMBLE, release.87 (Gallus_gallus.Gallus_gallus-5.0.dna.toplevel.fa and Gallus_gallus.Gallus_gallus-5.0.87.gtf). The aligned read counts were obtained using htseq-count as part of the package HTSeq-0.6.1.The Bioconductor package edgeR_3.1.2 with limma_3.22.7 was used to perform the differential gene expression analysis in R Studio v 1.0.143. The differentially expressed genes (DEGs) between the control group and carnosic acid group were identified at combined cut-offs with *p* value <0.05 and fold change>1.5. The DEGs were uploaded to DAVID Bioinformatics Resources (https://david.ncifcrf.gov/) online bioinformatics analysis system for functional annotation and canonical pathway analyses.

### GO enrichment analysis of differentially expressed genes

After gene annotation with DAVID-BR, Gene Ontology (GO) analysis (http://www.geneontology.org/page/go-enrichment-analysis) was applied to investigate the underlying functions (i.e., biological process, molecular function and cellular component). The *P* value denoted the significant of the GO term enrichment among differentially expressed genes (Adj *P*-value < 0.05).

### Real-time quantitative reverse transcription-PCR (qRT-PCR)

Key PCR primers applied to this study are listed in Table 8. Extracted RNA (200 ng) was used for cDNA synthesis using Thermo Scientific RevertAid First Strand cDNA synthesis kit (Cat#: K1621) according to the manufacturer instructions. The qPCR assays were performed using iTaq universal SYBR Green Supermix (Bio-Rad Laboratories, Hercules, CA, USA). Briefly, cDNA was diluted 10-fold, and 1 μL of each diluted sample was added to a 10-μL reaction mix containing 50 ng of forward and reverse primers of specific target gene. The β-actin house-keeping gene was used as an internal reference to normalize target gene transcript levels^54^. Reactions were conducted in triplicate. The threshold cycle (C_T_) values for genes of interest were normalized to an average C_T_ value of the house-keeping genes and the relative expression of replicate was calculated by applying the 2^-ΔΔCt^ method^55^.

**Table 8:** qRT-PCR target genes and their oligonucleotide primers applied in this study.

Target Gene	Primer Sequence (5′-3′)	Product size (bp)
*AKR1D1*	F: TCCACCAGAGCTGGTACGTC	319
	R: ATAGGGATGGCATTCAACCTGG	
*CYP3A80*	F: AATGGGACTCCTTCCAGACCT	235
	R: CCTGCCATCATAAATCCCCC	
*GSTA3*	F: AATTTCCCCTCTTGCAGAGTT	194
	R: CACTCCGCTTATCAGCAAAC	
*S100A9*	F: TCGCTGCTTCACCAACATGA	201
	R: ACCTGATTCTTCACGTGCTTCA	
*AvBD6*	F: TCCAGGGTGTTGCAGGTCAG	122
	R: ACTGCCACATGATCCAACCC	
*G0S2*	F: CGGCCTCAGAACGGAGC	113
	R: TCACCATCTTCCTGTTGGGC	
*GSTA3*	F: AATTTCCCCTCTTGCAGAGTT	194
	R: CACTCCGCTTATCAGCAAAC	
*ABCG1*	F: GAGCTGGAGCCGTCGGTAG	121
	R: CAGAATTGCTGGCGTTCAGAGC	
*ACER2*	F: CTGGTGCGAGGACAACTACA	183
	R: ACAGATCCAATTCCGACCACAA	
*CCR10*	F: CTGTGCAATGGAGGAGGCAAAC	82
	R: CGAGATGCTGTAGTCCCAGG	
*β-actin*	F: CTCTGACTGACCGCGTTACT	172
	R: TACCAACCATCACACCCTGAT	

### Statistical analysis

Chicken BW, BW gain, FCR, the levels of serum biomarkers (glucose, total cholesterol, triglycerides, VLDL) were compared among treatments using mixed model analysis of variance procedure. Analyses were conducted with SAS v9.4 (SAS Institute, Cary, NC, USA) and least squares means were compared at the significance level of *P* less than 0.05. BA analysis was performed using Waters MassLynx Software V4.2 SCN943 (Waters Corporation, USA). Prism 7.0 (GraphPad Software Inc., La Jolla, CA) was applied for graphic construction. For heatplot generation data was plotted using RStudio software version 1.1.453. Statistical significance for BA samples was calculated using using Kruskal wallis: $*P* < 0.05; $$*P* < 0.01; $$$*P* < 0.001 followed by Mann Whitney t-Test (2-tailed): *P < 0.05; **P < 0.01; ***P < 0.001.

## Supplementary information

Supplementary Information.

## Data Availability

RNA-Seq data generated for this study have been deposited in the National Center for Biotechnology and Information (NCBI) Sequence Read Archive (SRA) with accession number SRP149780.
