# Supplementary material for: Evaluation of bile salt hydrolase inhibitor efficacy for modulating host bile profile and physiology using a chicken model system
Source: Sci Rep. 2020 Mar 18;10:4941. doi: 10.1038/s41598-020-61723-7 (PMC7080769; doi:10.1038/s41598-020-61723-7)
Supplement: Supplementary file 1 — Supplementary Information. [file 41598_2020_61723_MOESM1_ESM.pdf]

SUPPLEMENTAL INFORMATION

Evaluation of bile salt hydrolase inhibitor efficacy for modulating host bile profile and physiology using a chicken model system

Wenjing Geng[$1], Sarah L. Long[$2,3], Yun-Juan Chang[4], Arnold M. Saxton[1], Susan A. Joyce*[2,3], Jun Lin*[1]

[1]Department of Animal Science, The University of Tennessee, 2506 River Drive, Knoxville, USA

[2]APC Microbiome Ireland, University College Cork, Cork, Ireland

[3]School of Biochemistry and Cell Biology, University College Cork, Cork, Ireland

[4]Department of High Performance Computing and Research, University of Rutgers, Newark, USA

[$]These authors contributed equally to this work

Running title: Efficacy of bile salt hydrolase inhibitors

Corresponding authors:
Dr. Jun Lin
Department of Animal Science
The University of Tennessee
2506 River Drive
Knoxville, TN 37996-4574
Phone: (865) 974-5598. Fax: (865) 974-7297
E-mail: jlin6@utk.edu

Dr. Susan A. Joyce
APC Microbiome Ireland
University College Cork
Cork, Ireland T12 YT20
Phone: 021 4901343
E-mail: S.Joyce@ucc.ie

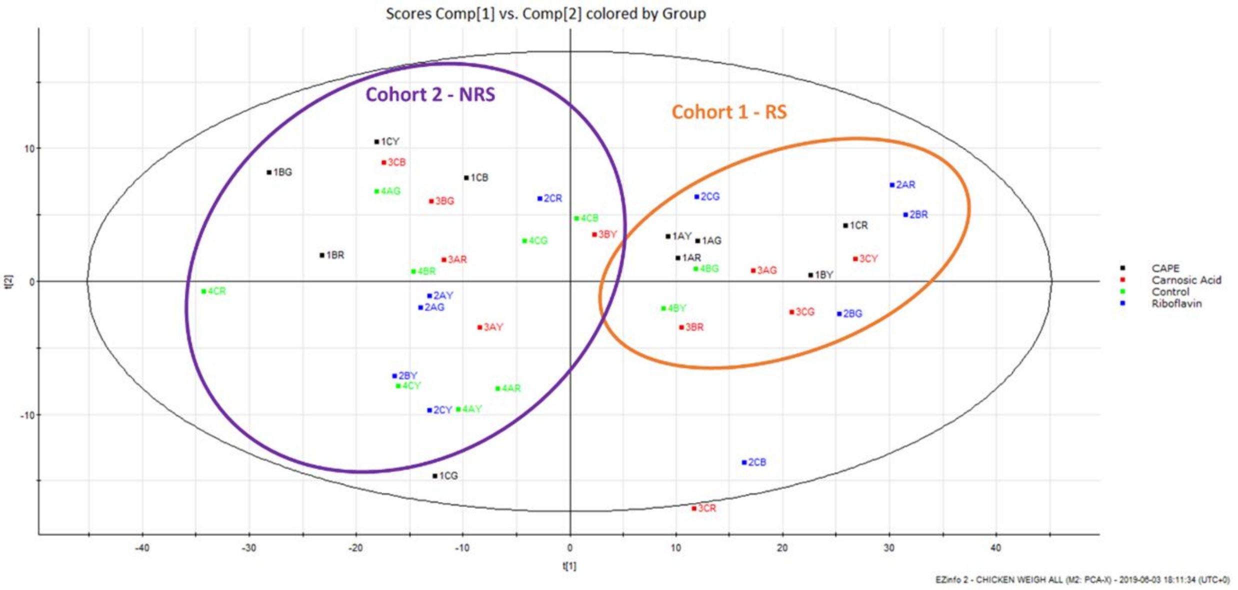

Figure S1. Broiler chicken body weight PCA analysis. Samples clustered into two distinct cohorts. Each point represents an individual chicken which is labelled by their corresponding study ID. Further analysis revealed that animals in cohort 1 displayed higher body weights and were designated treatment responders (RS). Cohort 2 were designated treatment non-responders (NRS) which contained animals from the Control group. PCA plot was generated using EZInfo software from Waters™.

Table S1. RNA sample description and mapping statistics to reference genome.

Tag name	Treatment [a]	Concentration (ng/µL)	RIN	28S/18S	Number of reads in pair (raw)	Number of reads in pair (Q trimmed)	Mapping Rate (%)	Non-specific mapping of mapped (%)
**Liver**								
LT1	T	320	8.6	1.9	26,343,666	24,990,680	86.4	3.4
LT2	T	2,664	9.4	2.1	37,647,864	35,569,752	86.7	3.6
LT3	T	4,328	9.2	1.9	9,188,235	8,510,958	85.0	3.3
LT4	T	848	9.2	1.9	90,545,141	84,666,914	68.9	3.4
LC1	C	3,576	9.2	2.1	27,499,176	25,823,234	88.2	3.5
LC2	C	3,216	9.1	2.0	14,620,336	13,514,440	86.4	3.5
LC3	C	4,760	9.1	2.1	36,029,297	34,089,068	85.8	4.1
LC4	C	4,912	9.3	2.2	134,362,985	128,352,800	64.8	3.8
**Ileum**								
IT1	T	2,088	9.5	2.0	14,406,611	13,461,675	82.8	4.18
IT2	T	3,424	9.6	2.2	14,867,215	14,004,769	83.4	4.0
IT3	T	2,320	9.4	2.0	20,300,211	18,961,626	84.1	4.3
IT4	T	1,460	8.9	2.0	29,154,179	27,535,357	82.6	5.5
IC1	C	2,068	9.0	1.8	18,346,963	17,380,507	83.7	7.9
IC2	C	1,524	9.3	1.9	162,762,428	155,661,706	61.2	4.0
IC3	C	1,476	8.6	1.8	35,944,368	33,371,893	82.8	4.6
IC4	C	320	8.9	1.8	13,162,436	12,258,294	82.2	4.0

[a]T stands for treatment; C stands for control.
